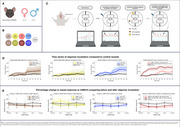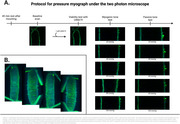# Middle Cerebral Artery Remodelling as a Response to Alzheimer's Disease Proteins

**DOI:** 10.1002/alz70855_097313

**Published:** 2025-12-23

**Authors:** Maria Hovmann Andresen, Eugenio Gutiérrez‐Jiménez, Vladimir Matchkov

**Affiliations:** ^1^ Aarhus University, Aarhus, Midtjylland, Denmark; ^2^ CFIN, Department of Clinical Medicine, Aarhus University, Aarhus, Denmark; ^3^ Aarhus University, Aarhus, Denmark

## Abstract

**Background:**

Amyloid‐beta (Aβ) oligomers and tau fibrils, both involved in the pathophysiology of Alzheimer's Disease, accumulate inside the walls of pial vessels. However, their effect on cerebral vascular tone and functionality remains incompletely understood.

**Method:**

We measured functional changes in middle cerebral arteries (MCA) from wild‐type mice (C57Bl/6NRJ) (*n* =  42) (Figure 1A) using a wire myograph (Figure 1C). MCAs were exposed to various concentrations of Aβ oligomers (C_aβ40_, C_aβ42_: 1 nM and 1 µM) and tau fibrils (C_tau_: 5 nM and 50 nM) for one hour (Figure 1B). Before and after incubation, we assessed the vessel's contractility using cumulative concentrations of U‐46619 (30 nM – 3 µM) and evaluated relaxation responses to the carbonic anhydrase inhibitor acetazolamide (ATZ) in various concentrations (1 mM ‐ 10 mM) on vessels pre‐constricted with U‐46619 (Figure 1C).

**Results:**

Aβ40 oligomers and tau fibrils significantly increase MCA baseline tension (Figure 1D) and, in combination with Aβ42, caused a marked attenuation in vasoconstrictive responses to U‐46619 (Figure 1E), suggesting a synergistic effect on vascular dysfunction.

**Conclusion:**

This study demonstrates that tau fibrils induce vascular dysfunction, which has previously been described for Aβ oligomers. The combined effect of these proteins, resembling a more comprehensive model of patient pathology, highlights the importance of targeting vascular disturbances in Alzheimer's Disease and provides insights into potential mechanisms affecting cerebral blood flow during disease progression. We have initiated imaging of vessels mounted in a pressure myograph using a two‐photon to examine vascular changes, particularly focusing on structural changes. The protocol with preliminary data is attached in Figure 2.